# Neurotoxicity-Based Toxicometabolomics of *N*‑Ethyl Pentedrone Using Zebrafish as an In Vivo Model

**DOI:** 10.1021/acsomega.5c08710

**Published:** 2025-10-18

**Authors:** Alexandre B. Godoi, Leonardo C. Rodrigues, Matheus F. Alves, Viviane C. Fais, Claudia V. Maurer-Morelli, Jose L. Costa

**Affiliations:** † Campinas Poison Control Center, 28132 Universidade Estadual de Campinas (UNICAMP), Campinas 13083-888, SP, Brazil; ‡ School of Medical Sciences, Universidade Estadual de Campinas (UNICAMP), Campinas 13083-894, SP, Brazil; § Leibniz Institute of Vegetable and Ornamental Crops (IGZ), Theodor-Echtermeyer-Weg 1, Großbeeren 14979, Germany; ∥ Institute of Biodiversity, Ecology, and Evolution (IBEE), Friedrich Schiller University Jena, Dornburgerstraße 159, Jena 07743, Germany; ⊥ Laboratoty of Zebrafish, School of Medical Sciences, Universidade Estadual de Campinas (UNICAMP), Campinas 13083-894, SP, Brazil; # Faculty of Pharmaceutical Sciences, Universidade Estadual de Campinas (UNICAMP), Campinas 13083-871, SP, Brazil

## Abstract

New psychoactive substances (NPS), particularly synthetic cathinones, have gained increasing attention due to their widespread recreational use and associated public health risks. Within this class, *N*-ethyl pentedrone (NEP) has been linked to cases of severe intoxication; however, its metabolism and neurobiological effects have remained poorly characterized. This study aimed to investigate the metabolism and neurotoxicological effects of NEP using the Zebrafish Water Tank protocol, an established alternative model for evaluating the toxicological properties of psychoactive substances. Liquid chromatography-high-resolution mass spectrometry (LC-HRMS) was employed to characterize NEP metabolites in exposure water and zebrafish brain tissue, complemented by a toxicometabolomic approach to elucidate adverse events triggered in the central nervous system. The analysis revealed metabolic processes of NEP primarily through *N*-dealkylation, β-ketone reduction, hydroxylation, and *O*-glucuronidation. Metabolites were identified in exposure water (*n* = 3) and in brain tissue (*n* = 7). Untargeted toxicometabolomics revealed six statistically significant differentially expressed metabolites between the exposed and control animals. Four annotated metabolites were found upregulated in NEP-exposed zebrafish: propionylcarnitine (*p* = 0.001, fold change (FC) = 2.2), l-kynurenine (*p* = 0.024, FC = 2.9), adenylyl(3′–5′)­cytidine (*p* = 0.027, FC = 2.1), and cytidine (*p* = 0.028, FC = 2.5), whereas two were downregulated: putatively PI­(19:1­(9Z)/0:0) (*p* = 0.032, FC = 0.2) and an identified compound (*p* = 0.034, FC = 0.3). Altogether, these findings suggest neurochemical alterations induced by NEP exposure involving disruptions in neurotransmitter biosynthesis and function, energy metabolism, and oxidative stress responses. Furthermore, changes in lipid metabolism and mitochondrial function highlight the potential mechanisms underlying the observed neurotoxicity. Overall, our findings provide new insights into the metabolism and neurobiological effects of NEP, underscoring its potential neurotoxicity and associated mechanisms. Additionally, this study reinforces the utility of zebrafish as a model for investigating the pharmacokinetics and toxicodynamics of NPS.

## Graphical abstract

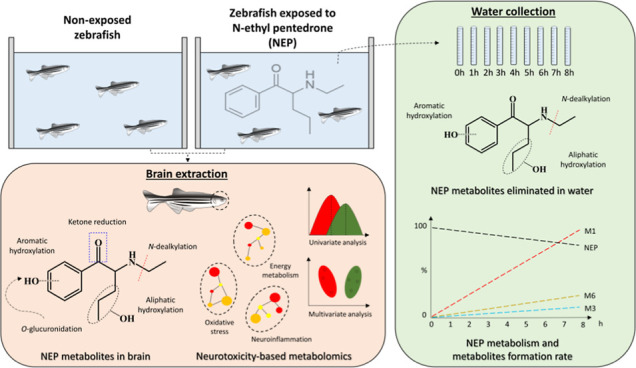

## 1 Introduction

In recent decades, the emergence of substances intentionally synthesized to circumvent existing legislation has raised significant concerns among authorities, healthcare systems, and toxicologists.^1^ New psychoactive substances (NPS) are defined as substances with central nervous system (CNS) activity that are not regulated by the 1961 Convention on Narcotic Drugs or the 1971 Convention on Psychotropic Substances.^2^ The number of NPS has increased substantially over the years, necessitating a systematic and thorough classification. Consequently, different classes of NPS have been established based on their molecular structures.^3^ Among these classes, synthetic cathinones constitute one of the most numerous and widespread groups within NPS.^4^ Only in the metropolitan region of Campinas, 15 cases of synthetic cathinone-related intoxications were recorded by the Campinas Poison Control Center (Campinas, Sao Paulo, Brazil) between 2016 and 2021, underscoring the prevalence and significance of these substances.^5^

In this context, the 2010s were marked by the predominance of synthetic cathinone seizures and reports of a new representative of this group, *N*-ethyl pentedrone (NEP), which emerged primarily by the internet.^6^ This molecule is an *N*-ethyl-substituted derivative of the widely known pentedrone, which is related to seizures reported by entities such as the Center for Forensic Science Research and Education (CFSRE) in the USA,^7^ and the police forces of the Northeastern states of Brazil.^8^ NEP has been linked to five cases of intoxication in recent years, all reaching severe outcomes.^9–13^ Notably, in four of these cases, NEP was found in combination with other substances, including NPS or ethanol, suggesting that potential drug–drug interactions may have contributed to the severity of these incidents. A recent pharmacokinetic study using human liver microsomes (HLM) indicated that NEP has a prolonged in vitro elimination half-life, suggesting slow metabolic clearance.^14^ These findings highlight the need for further research on NEP’s pharmacokinetics and toxicity, especially in the context of poly drug use. Despite the evident risks associated with NEP exposure, few studies have investigated its toxicity.

Establishing parameters related to the behavior of xenobiotics in biological systems—from their entry into the organism to the manifestation of their pharmacological activity and elimination—is essential for studies aimed at discovering new drugs.^15^ However, when applied to the context of drugs of abuse, evaluating these parameters is necessary to understand specific aspects of these substances in the body, such as clinical signs of intoxication, as well as to propose treatment strategies and develop analytical methods for detecting the drugs and their metabolites.^16^ Therefore, assessing the metabolism of such molecules is crucial for predicting properties related to their effects and providing parameters regarding their activities, interactions, toxicities, and potential treatments.

The use of animal models represents a powerful approach for determining the metabolic properties. These in vivo methods provide a comprehensive understanding of the organism and allow the assessment of physiological factors that can influence kinetics and metabolite production.^17^ Various animals have been employed in such studies, with rodents being the traditional and most used model.^18^ However, alternative models are increasingly replacing rodents in research. The zebrafish (*Danio rerio*) is a well-established vertebrate model that shares significant genetic and functional similarities with mammals. Its unique features, such as rapid development and optical transparency, make it ideal for high-throughput experiments.^18,19^

The Zebrafish Water Tank (ZWT) protocol has proven to be a suitable method for evaluating various kinetic processes including absorption, distribution, metabolism, and excretion. This methodology not only allows for quick and robust inferences concerning the identification of metabolite structures and their production ratios but also enables reliable extrapolations about human metabolism, given the notable enzymatic orthology shared between both species.^18,20^ Prado et al. (2021) demonstrated the applicability of the ZWT model in evaluating the metabolism of synthetic cathinones, highlighting important and remarkable features of these substances.^21^

In conjunction with kinetic assessments, elucidating the mechanisms of action of xenobiotics provides crucial information for understanding their toxicities. Metabolomics has been applied to a wide range of studies as endogenous small metabolites are related to numerous cellular biochemical events.^22^ Consequently, combining metabolomic approaches with toxicological assessments has expanded and enhanced the ability to identify potential exposure biomarkers, propose mechanisms of toxicity, and even suggest prospective antidotes.^23^ Toxicity-based metabolomics, or toxicometabolomics, encompass qualitative and quantitative analysis of a broad range of substances within various biological matrices, such as blood plasma, urine, and tissues.^23^ For such comprehensive analytical coverage, highly sensitive and robust instruments are employed, with liquid chromatography-high resolution tandem mass spectrometry (LC-HRMS) and nuclear magnetic resonance being extensively used.^23,24^

Herein, this study aimed to characterize the metabolism and neurological effects of a substance frequently detected in forensic cases and strongly associated with severe intoxications using an in vivo zebrafish model. Specifically, we described the dynamics of metabolite production in exposure water and brain tissue and performed an LC-HRMS-based toxicometabolomic evaluation of zebrafish brains, identifying potential metabolites associated with NEP-induced neurotoxicity.

## 2 Results

### 2.1 NEP Metabolism in Zebrafish Brain Tissues and Exposure Water

Throughout the entire experiment, no relevant behavioral or physical alterations were observed in the animals, and all subjects survived through drug exposure. Although these findings provide valuable insights into NEP metabolism, the study was not specifically designed or structured to assess behavioral outcomes.

To confirm the absence of NEP in nonexposed animals, negative controls were analyzed. Neither NEP nor its metabolites were detected in these samples (data not shown). This evaluation was performed using XIC from full-scan acquisitions, considering the exact masses of NEP and its metabolites and ensuring the absence of these substances. Additionally, a positive control was included to assess the stability of NEP in Reconstituted Water (RW) and to identify potential nonenzymatic reactions throughout the experiment. In Supporting Information, Figure S1A illustrates the NEP/IS area ratio over an 8 h period in the absence of zebrafish and under experimental conditions. Nonlinear regression analysis of these measurements indicated that the NEP remained highly stable throughout the exposure period. Furthermore, NEP metabolism in the exposure tanks was assessed and demonstrated in Figure S1B. NEP/IS area ratio was measured in triplicate over the 8 h experiment, allowing the calculation of mean values, standard deviations, and an evaluation of its decay profile using nonlinear regression.

### 2.2 Identification of NEP Metabolites in Zebrafish Brain Tissues and Exposure Water

NEP metabolites were analyzed, and their formation dynamics monitored in exposure water throughout the experiment. Additionally, metabolites were examined in zebrafish brain tissues aiming to detect and characterize the presence of NEP and metabolites in the brain, given its important activity in CNS.^25^ All identified metabolites satisfied the identification criteria established. Only metabolites 7 and 8 (M7 and M8) presented fragments with a mass error greater than 15 ppm (−25.67, −25.98, and 32.70 ppm for fragments F10, F12, and F14 in M7, respectively, and 27.18 ppm for fragment F14 in M8). Nevertheless, these fragments were maintained in the analysis as previous studies demonstrated similar fragmentation patterns for other synthetic cathinones.^26^ The mass spectra of the identified substances were displayed for zebrafish brain tissues and exposure water in the Supporting Information (Figures S2 and S3, respectively). The molecular structures of the fragments were also detailed in Figure S4, following fragmentation reactions previously described.^14,26,27^ Additionally, analytical parameters regarding NEP and metabolite identification, such as molecular formula, theoretical and measured exact mass, mass error, and retention time (RT), were described for both approaches in Table S1 in Supporting Information.

The metabolites identified from both sources were also organized into a metabolic pathway illustrating the reactions involved in NEP metabolism, as demonstrated in Figure 1. Four phase I reactions were observed: *N*-dealkylation, β-ketone reduction, aromatic hydroxylation, and aliphatic hydroxylation. Only *O*-glucuronidation was observed as a phase II reaction.

**Figure 1:**
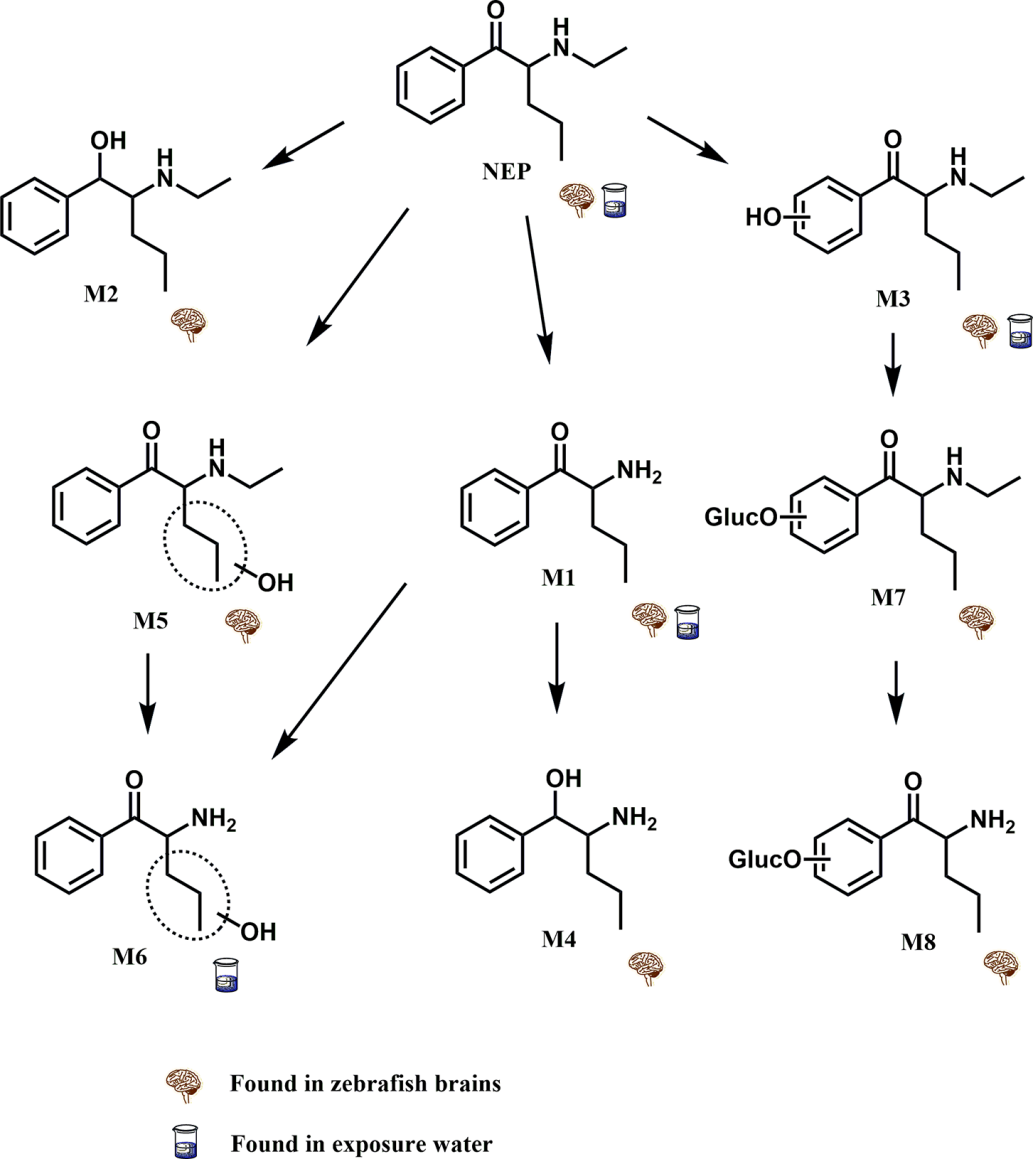
NEP metabolic pathway identified in zebrafish brain and exposure water after 8 h exposure.

#### 2.2.1 Phase I NEP Metabolites

Metabolite 1 (M1) was the most abundant metabolite in both zebrafish brain and exposure water, characterized by an oxidation reaction of *N*-dealkylation. Metabolite 2 (M2) was formed by a reduction of NEP’s β-ketone, generating an alcoholic derivative metabolite, which was slightly detected in zebrafish brains and not detected in exposure water. Similarly, M1 also underwent a β-ketone reduction, producing metabolite 4 (M4)–the second most abundant metabolite in zebrafish brains. Another metabolic reaction observed for M1 was a side chain aliphatic hydroxylation, producing metabolite (M6), which only reached a maximum NEP-relative abundance of 0.1%. Hydroxylation reactions in the parent compound were also observed in zebrafish, occurring in the aromatic ring (M3) and in its alkyl side chain (M5). M3 was observed in both sources evaluated in this study, while M5 was only found in cerebral tissue. Despite the differences in hydroxylated metabolites, both were identified in low amounts.

#### 2.2.2 Phase II NEP Metabolites

Conjugated metabolites were also observed, all produced after an aromatic hydroxylation, highlighting the importance of phase I metabolism in functionalization steps that allow the substance to conjugate with electrophilic biomolecules such as uridine diphosphate glucuronic acid and 3′-phosphoadenosine 5′-phosphosulfate (PAPS). The two phase II metabolites identified were products of glucuronide conjugation: M7 was produced by an aromatic hydroxylation followed by *O*-glucuronidation and M8 by *N*-dealkylation of M7. These two metabolites were exclusively observed in zebrafish brains, as the exposure water group sample preparation included enzymatic hydrolysis with β-glucuronidase. M7 and M8 were the fourth and third most abundant metabolites in zebrafish cerebral tissues, respectively, underscoring the importance of phase II reactions in NEP metabolism. In Figure 2A, the time-course formation of NEP metabolites in the exposure water was shown as the ratio of the metabolite peak area to IS peak area. The production of M1 was clearly dominant, while M3 and M6 were present at much lower levels. Therefore, Figure 2B presents a close-up view of M3 and M6, highlighting their subtle formation patterns throughout the exposure period. The abundance of metabolites in zebrafish brain tissue is demonstrated in Figure 3 as the metabolite/IS absolute area ratio. The Venn diagram demonstrated in Figure 4 illustrates the comparison between the previously identified in vitro (RLM, MLM, and HLM) and the in vivo NEP metabolites found using zebrafish models (Zebrafish brain and ZWT).^14^

**Figure 2:**
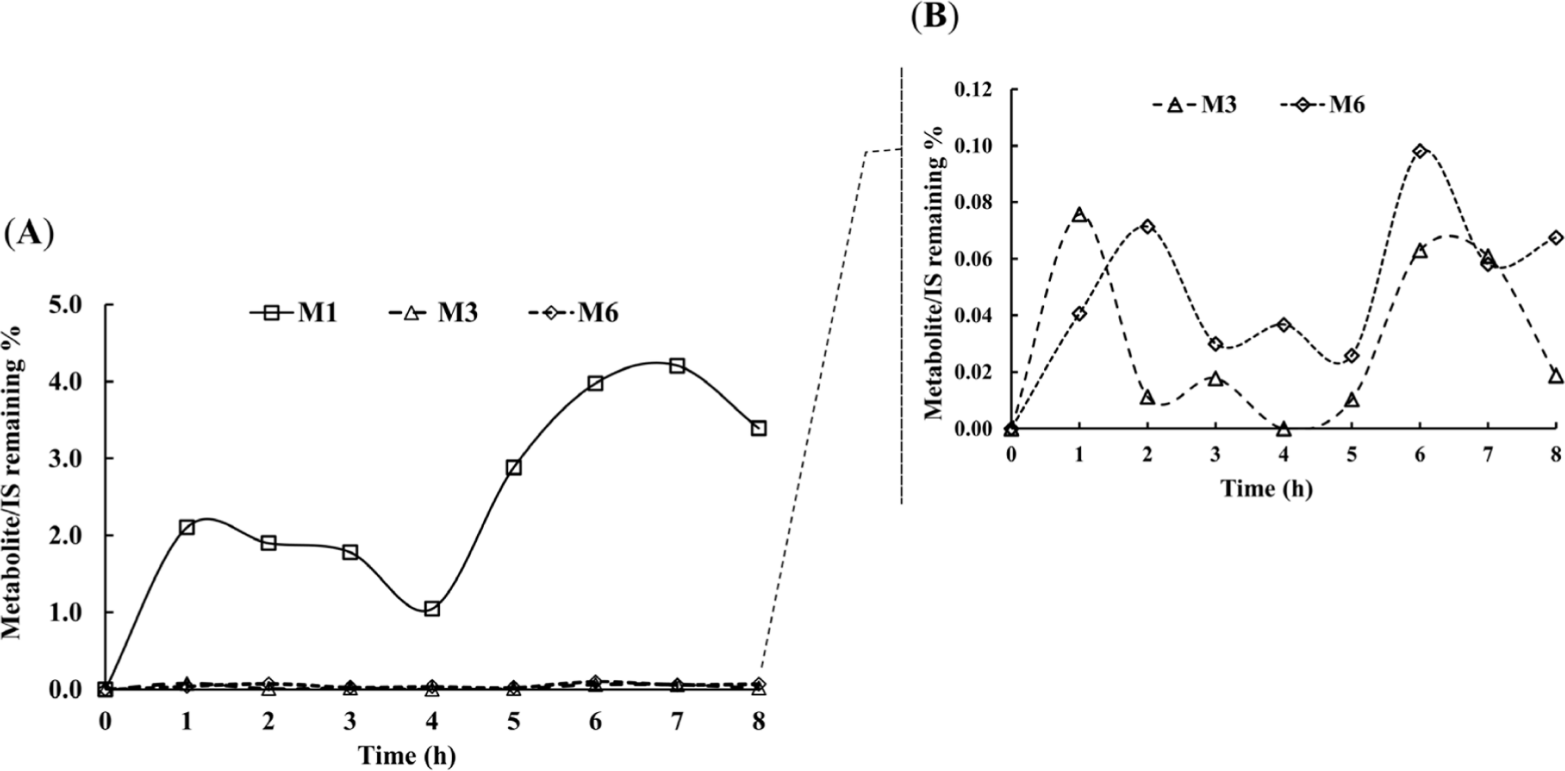
Formation rate of NEP metabolites in exposure water, expressed as the ratio of metabolite peak area to internal standard (IS) peak area. (**A**) Metabolite formation over time for M1, M3, and M6 and (**B**) a detailed representation of M3 and M6 formation.

**Figure 3:**
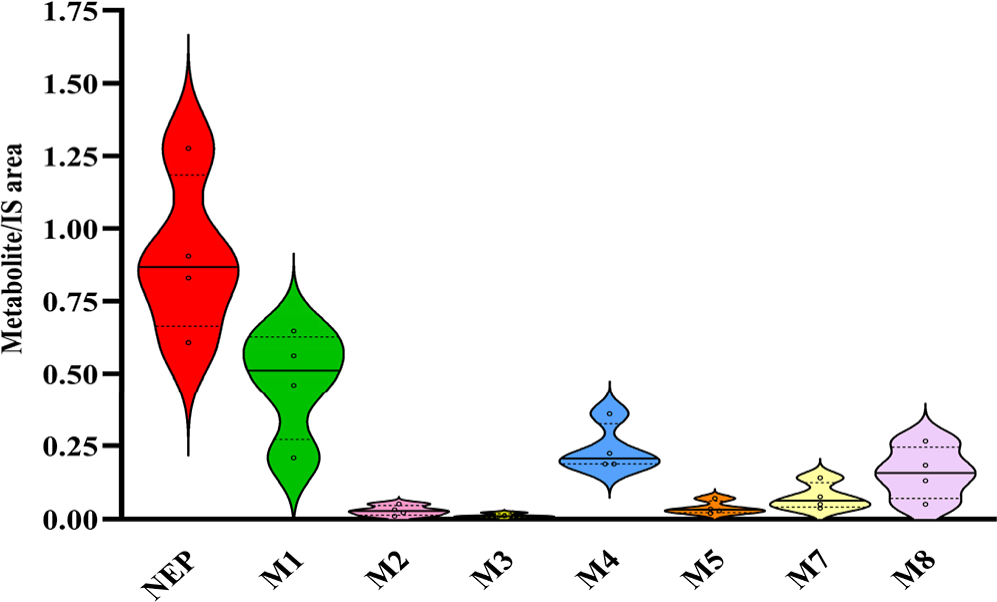
Abundance disposition of NEP and the identified metabolites found in zebrafish brains after 8 h of exposure.

**Figure 4:**
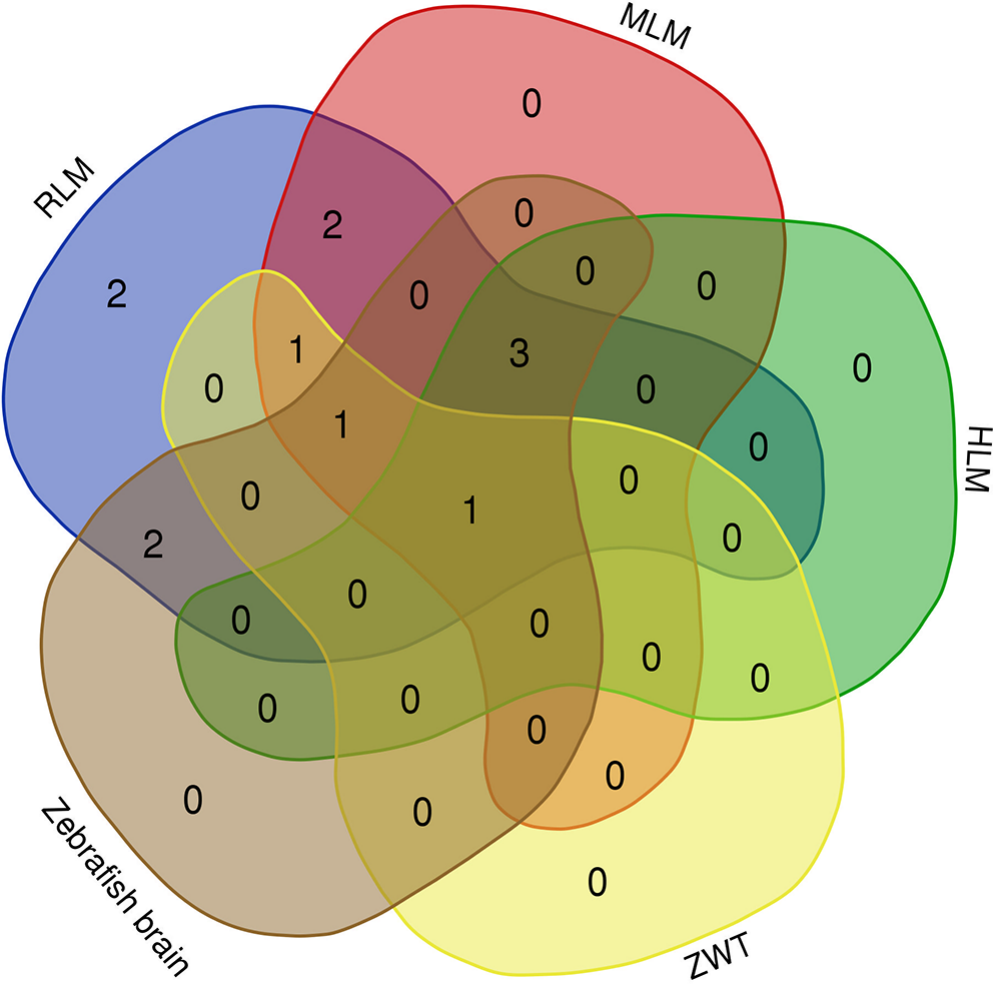
Venn diagram comparing the number of NEP metabolites identified through in vitro models using rat (RLM), mouse (MLM), and human (HLM) liver microsomes, and in vivo models using zebrafish exposure water from ZWT experiment and zebrafish brain tissues.

### 2.3 NEP Neurotoxicity-Based Toxicometabolomics

The obtained matrix after data curation and filtering procedures resulted in 4027 features related to four groups (blank, quality control (QC), exposed, and control animals). Blanks and the six QCs injected before samples were removed from the data set before any statistical analysis. The robustness and performance were assessed by evaluating the QC samples using Principal Component Analysis (PCA) analysis. This unsupervised approach facilitates a comprehensive investigation highlighting the reliability and accuracy of the samples analyzed in this study. The chromatographic system was appropriately equilibrated after six injections of QC, as demonstrated in Figure 5A. Additionally, our analysis did not demonstrate considerable group clustering through PCA analysis (Figure 5B). To elucidate the metabolomic differences between exposed and nonexposed animals to NEP, an Orthogonal Partial Least Squares Discriminant Analysis (OPLS-DA) model was constructed, as shown in Figure 5C. This supervised method demonstrated discrimination between these groups, indicating potential alterations in the metabolomic profile resulting from NEP exposure. The results indicated values for *R* ^2^ of 0.69 and for *Q* ^2^ of 0.30 in the cross-validation test performed using leave-one-out cross-validation (LOOCV) as demonstrated in Figure 5D.

**Figure 5:**
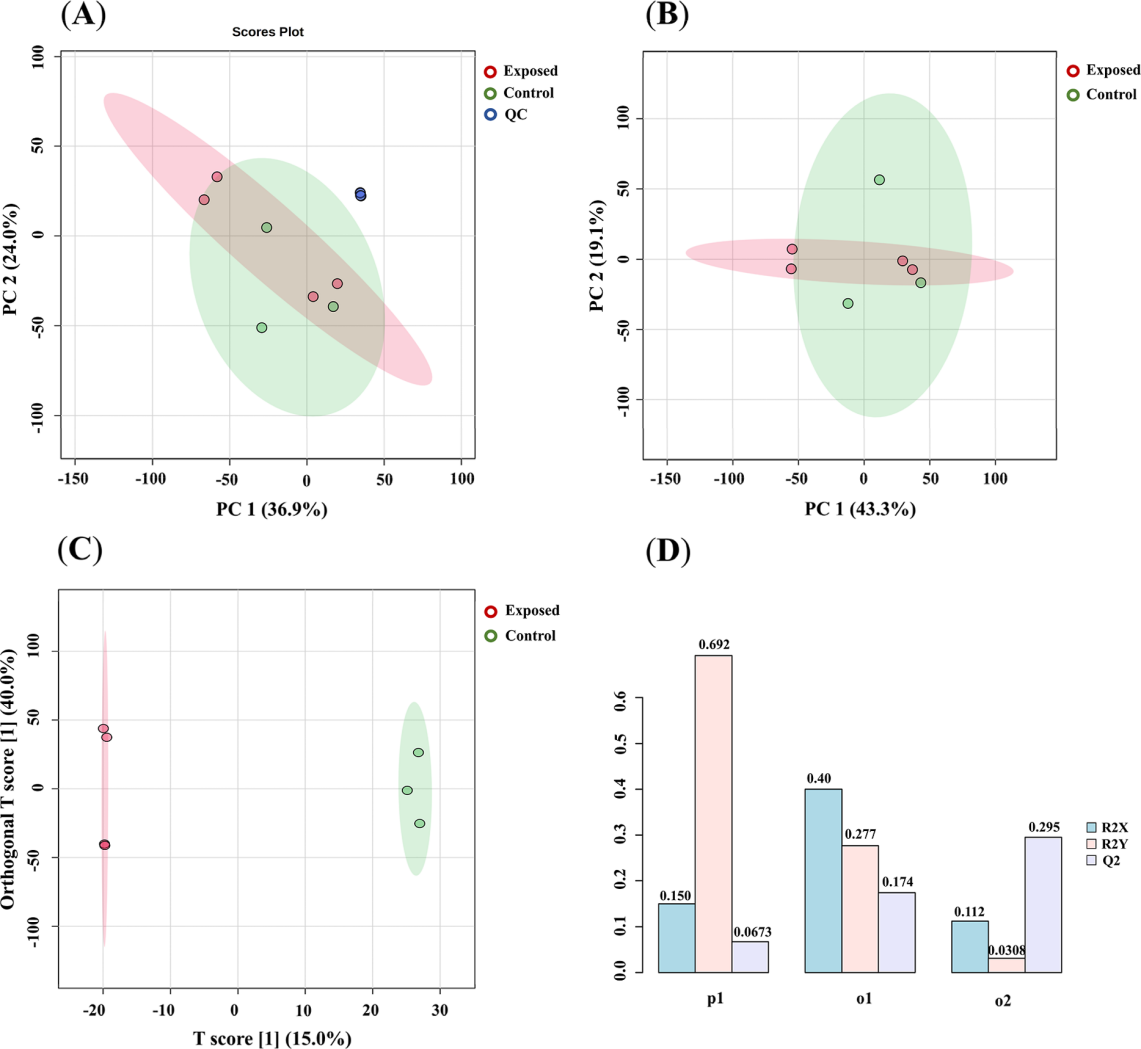
Multivariate statistical analysis of the metabolomic data set. (**A**) PCA score plot demonstrating the clustering of exposed (red), control (green), and QC (blue) samples. (**B**) PCA score plot showing the distribution of exposed (red) and control (green) groups. (**C**) OPLS-DA score plot displaying distinct metabolic profiles between exposed (red) and control (green) groups. (**D**) Model validation parameters, including R2X, R2Y, and *Q* ^2^ values for the supervised model using leave-one-out cross-validation (LOOCV).

From the 4027 detected features, 611 were tentatively annotated as metabolites and lipids in different levels of confidence accordingly with the current reports’ recommendation.^28,29^ The majority could be annotated with level 2 of confidence by integrating MS-DIAL, MS-FINDER, HMDB, and Mass Bank libraries for MS2 spectral similarity analysis. Univariate analysis identified six statistically significant and differentially expressed features considering a *p*-value <0.05 and 0.5 ≤ fold change (FC) ≥ 2.0, as shown in the volcano plot in Figure 6A. Associating a VIP score cutoff of 2.0 from OLPS-DA model, all features highlighted by univariate analysis satisfied the established criteria. Values obtained by the area under the curve (AUC) of the receiver operator characteristics (ROC) analysis of these variables were also investigated in order to determine their prediction power (Figure 6B). Interestingly, four of these important features were found upregulated, while two were found downregulated. The upregulated features were annotated as metabolites such as cytidine, propionylcarnitine, l-kynurenine, and adenylyl(3′–5′)­cytidine. The apparent downregulated features were not annotated by spectral comparison, but one potentially corresponds to a glycerophosphatidylinositol derivative, tentatively annotated as PI­(19:1­(9Z)/0:0) by monoisotopic mass match, which can be attributed as an annotation at level 3 of confidence. The other downregulated feature (*m*/*z* 520.3333, RT 3.05) corresponds to an unknown metabolite, corresponding to an annotation at level 4 of confidence. Metabolites annotation and their statistical parameters are demonstrated in Table 1.

**Figure 6:**
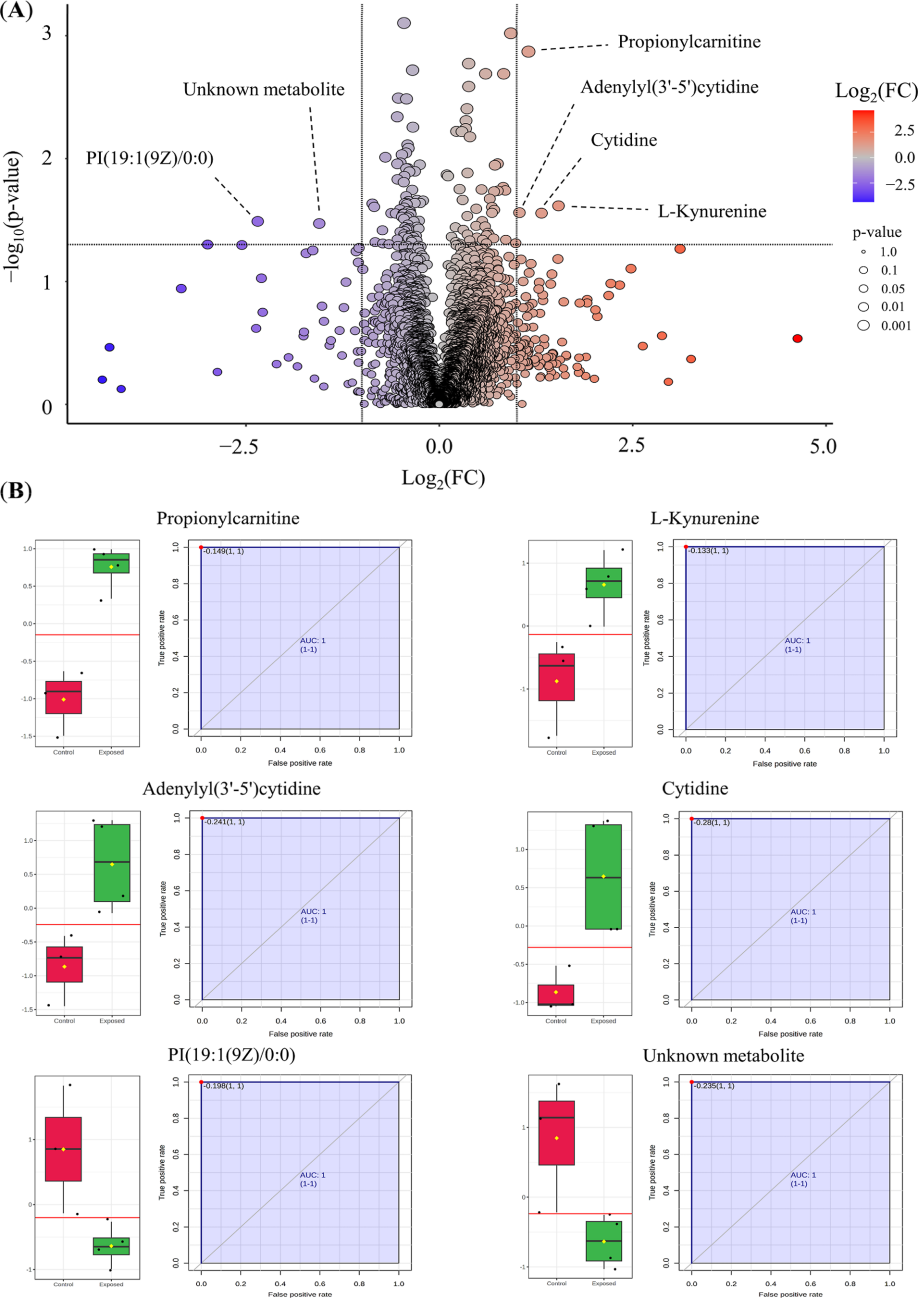
Univariate and biomarker analysis of differentially expressed metabolites. (**A**) Volcano plot displaying the differentially expressed metabolic features between exposed and control groups. Statistically significant features were identified based on *p*-value <0.05 and FC threshold of 0.5 ≤ FC ≥ 2.0, with upregulated metabolites highlighted in red and downregulated metabolites in blue. (**B**) Receiver Operating Characteristic (ROC) curve analysis illustrating the predictive power of the identified metabolites. The Area Under the Curve (AUC) values were calculated to assess the discriminative capacity of each feature.

**Table 1:** Annotation and Statistical Parameters of Differentially Expressed Metabolites^a^ ^b^

**metabolites annotation**									**univariate analysis**		OPLS-DA	**ROC analysis**
**metabolite**	**molecular formula**	**adduct**	**RT (min)**	**exact mass**	**theoretical adduct** *m*/*z*	**measured** *m*/*z*	**mass error (ppm)**	**ID level**	**fold change**	*p*-value	**VIP score**	**AUC**
propionylcarnitine	C_10_H_19_NO_4_	[M + H]+	1.98	217.1314	218.1392	218.1388	–1.8	2	2.2	0.001	2.4	1.00
l-Kynurenine	C_10_H_12_N_2_O_3_	[M + H]+	2.54	208.0848	209.0926	209.0924	–1.0	2	2.9	0.024	2.1	1.00
adenylyl(3′–5′)cytidine	C_19_H_25_N_8_O_11_P	[M + H]+	2.53	572.1380	573.1459	573.1457	–0.3	2	2.1	0.027	2.1	1.00
cytidine	C_9_H_13_N_3_O_5_	[M + H]+	0.97	243.0855	244.0933	244.0927	–2.5	2	2.5	0.028	2.1	1.00
PI(19:1(9Z)/0:0)	C_28_H_53_O_12_P	[M + H]+	3.10	612.3275	613.3353	613.3407	8.8	3	0.2	0.032	2.1	1.00
unknown metabolite		[M + H]+	3.05			520.3333		4	0.3	0.034	2.0	1.00

a Listed metabolites were identified when possible.

b For each annotated metabolite, molecular formula, identified adduct, RT, theoretical and measured mass to charge ratio (*m*/*z*) along with the corresponding mass errors, and the identification level (ID level) based on reporting guidelines were shown.^28,29^ Univariate analysis results include FC and statistical significance using *t*-test (*p*-value), highlighting metabolites with *p* < 0.05 and 0.5 ≤ FC ≥ 2.0. OPLS-DA was performed, and Variable Importance in Projection (VIP) scores above 2.0 were considered relevant. Receiver Operating Characteristic (ROC) analysis was conducted to assess the discriminative power of the identified metabolites, with area under the curve (AUC) values indicating their predictive accuracy.

To elucidate the metabolic perturbations associated with NEP neurotoxicity, the differentially expressed metabolites were subjected to pathway enrichment analysis using Metaboanalyst 6.0. Enrichment analysis identifying associated pathways related to the differentially expressed and significant metabolites are demonstrated in Figure 7A, where the node size illustrates the pathway impact factor (larger nodes implies in greater biological relevance), and pathway’s *p*-value were plot based on their colors (hotter colors implies in greater statistical significance and lowest *p*-value). Furthermore, pathway analysis based on *D. rerio* Kyoto Encyclopedia of Genes and Genomes (KEGG) database was also plotted and represented in Figure 7B, which the *x*-axis demonstrates the pathway impact values derived from topological analysis, while the *y*-axis corresponds to the −log_10_(*p*-value) from the enrichment analysis. Metabolic pathways exhibiting both high −log_10_(*p*-value) values and substantial impact scores are considered the most significantly affected (upper right region of the plots). Predicted dysfunctional enzymes (Figure 7C) and associated disease signatures (Figure 7D) had their statistical significance plotted in the *x*-axis, and it was also correlated to nodes color intensity. Additionally, the node size represents the pathway impact factor, with larger nodes signifying higher biological relevance.

**Figure 7:**
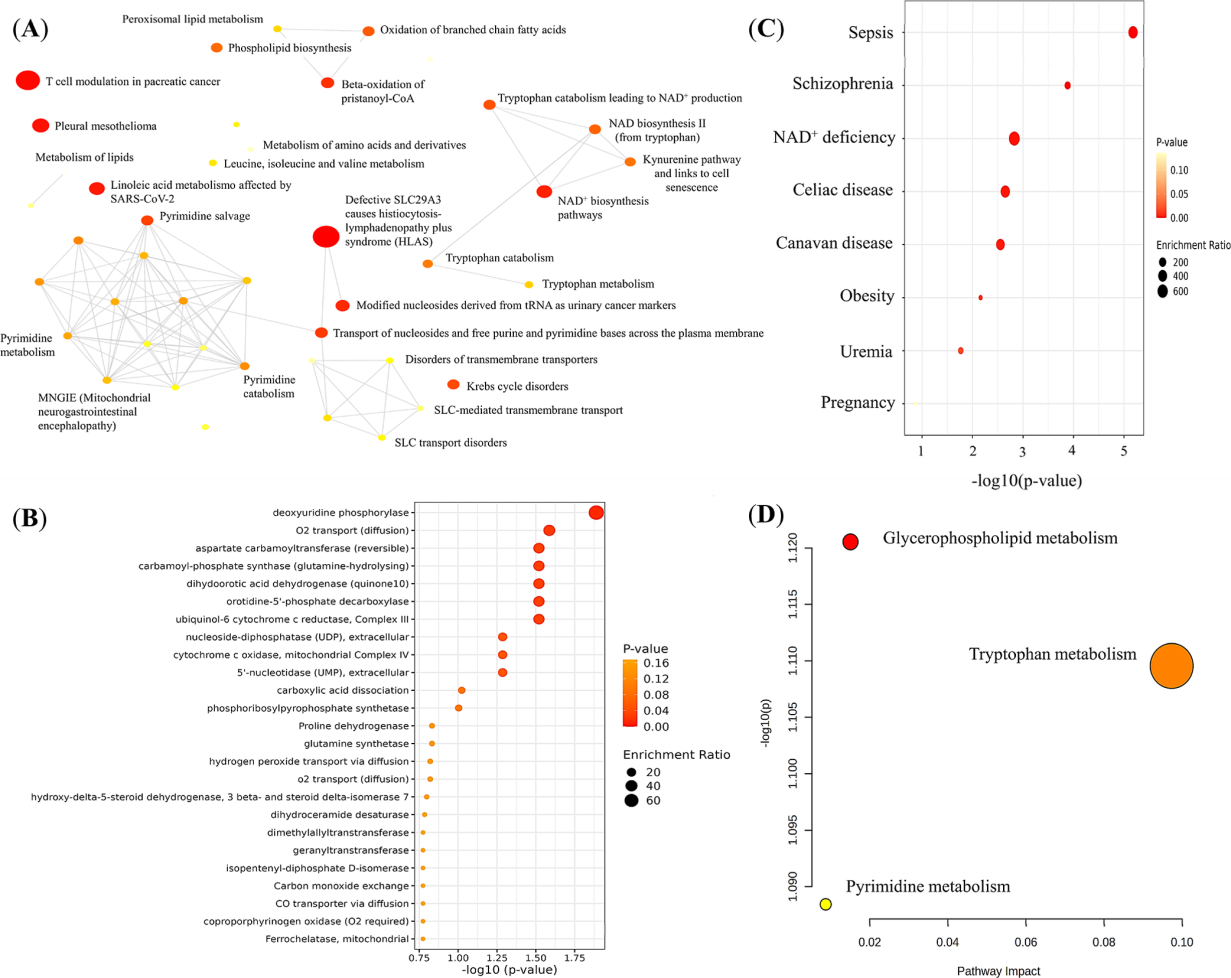
Metabolic pathways and associated with NEP neurotoxicity and enrichment analysis using the significantly altered metabolites. (**A**) Enriched metabolic pathways identified through pathway analysis (RaMP-DB), (**B**) pathway topology analysis based on *Danio rerio* KEGG database, (**C**) predicted dysfunctional enzymes associated with the altered metabolites, and the (**D**) disease signatures linked to NEP exposure.

Notably, pathways related to energy metabolism dysregulation such as NAD^+^ biosynthesis, beta-oxidation of pristanoyl-CoA, citric acid cycle disorders, leucine, isoleucine, and valine metabolism, oxidation of branched chain fatty acids, and mitochondrial neurogastrointestinal encephalopathy (MNGIE) were indicated. Neurotransmitter biosynthesis, degradation, and function pathways were also highlighted, such as tryptophan catabolism leading to NAD^+^ production, kynurenine pathway, and links to cell senescence, solute carrier (SLC) transport disorders, and disorders of transmembrane transporters. Additionally, inflammation (T cell modulation in pancreatic cancer), lipid homeostasis (phospholipid biosynthesis and peroxisomal lipid metabolism), and nucleotides metabolism (transport of nucleosides and free purine and pyrimidine bases across the plasma membrane, pyrimidine metabolism, nucleotide salvage, dihydropyrimidinase deficiency, and UMP synthase deficiency) were also found as enriched pathways. Other less or noncorrelated pathways were also found, such as defective SLC9A3 causes histiocytosis-lymphadenopathy plus syndrome, linolenic acid metabolism affected by SARS-CoV-2, modified nucleosides derived from rRNA as urinary cancer markers, and beta ureidopropionase deficiency. *D. rerio* KEGG pathway library also demonstrated the importance of amino acid metabolism (tryptophan metabolism), lipid metabolism (glycerophospholipid metabolism), and nucleotide metabolism (pyrimidine metabolism).

Disease-related plot illustrates the diseases most associated with the significantly altered metabolites, neurological (schizophrenia and Canavan disease), inflammatory (sepsis and celiac disease), and metabolic (NAD^+^ deficiency, obesity, and uremia) disturbances showing an enrichment ratio and statistical significance. These findings may indicate a direct link among metabolic dysregulation, neuroinflammation, and neurological dysfunction.

Finally, the findings related to key enzymes associated with the altered metabolites suggested disruptions in enzymatic activity linked to pyrimidine metabolism (deoxyuridine phosphorylase, UDP nucleoside-diphosphate, and UMP 5′-nucleotidase), energetic metabolism (O_2_ transport, ubiquinol-6 cytochrome *c* reductase, complex III, cytochrome *c* oxidase, mitochondrial complex IV, phosphoribosylphosphate synthetase, carbon monoxide exchange and transport, coproporphyrinogen oxidase, and mitochondrial ferrochelatase), amino acids metabolism (aspartate carbamoyltransferase, carbamoyl-phosphate synthase, carboxylic acid dissociation, proline dehydrogenase, and glutamine synthetase), and lipid homeostasis (hydroxy-delta-5-steroid dehydrogenase, 3-beta- and steroid delta-isomerase 7, dihydroceramide desaturase, and geranyltransferase, isopentyl-diphosphate d-isomerase), suggesting critical features for neuronal function and integrity.

Together, these results supply the hypothesized pathways and enzymes associated with dysregulation in energy metabolism, neurotransmitter production, neuroinflammation, nucleotide biosynthesis, and lipid homeostasis, potentially contributing to NEP-neurotoxicity mechanisms.

## 3 Discussion

The determination of NEP concentrations in exposure tanks revealed a slightly decreasing trend throughout the experiment. While this may suggest a kinetic profile, the exposure model used does not provide reliable information on the enzymatic kinetics of the substance in zebrafish.^21^ Nonetheless, we observed a sequential reduction in NEP levels at each measurement point, likely due to absorption and metabolism mechanisms, as there was no substantial decrease in NEP or formation of its metabolites in the positive control. Additionally, through XIC evaluations targeting NEP and metabolite exact masses, these substances were not found in negative controls. However, several metabolites were identified in exposure tanks along the parent compound, whose molecular structures were assigned through MS2 mass spectra. Measurements of the metabolite production rate and structural assignment in exposure tanks were performed in triplicate.

In total, eight NEP metabolites were identified in both exposure water and brain tissue. Seven metabolites were detected in zebrafish brains after 8 h of exposure, including two resulting from phase II *O*-glucuronidation (M7 and M8). In contrast, only three metabolites (M1, M3 and M6) were found in the exposure water, none from conjugation reactions. Notably, these metabolites exhibited a cumulative formation trend over successive incubation times. However, similar to the NEP decay, the formation behavior of these metabolites does not reliably characterize their kinetic profile. Since they can be excreted in water, they could be reabsorbed, subsequently metabolized, and then eliminated as another metabolite.

The formation profile showed that M1 was the most abundant metabolite in exposure water and also predominant in zebrafish brains, underscoring the importance of the *N*-dealkylation reaction in NEP metabolism. Previous work described NEP’s metabolic profile using a multispecies microsomal approach, where the *N*-dealkylated metabolite was also predominant in rat (RLM) and mouse (MLM) liver microsomes.^14^ Similarly, descriptions on the metabolism of other N-substituted synthetic cathinones have highlighted *N*-dealkylation reactions.^30,31^ The aromatic hydroxylated metabolite (M3) was identified in both water and brain analyses, but at lower amounts. Similarly, M2 and M5 were poorly detected in brain analysis, and not detected in water tanks, consistent with previous in vitro findings.^14^ Only M6 was absent in brain tissues but was found discretely in water tanks. M4, the second most abundant metabolite in zebrafish neural tissue, highlighted the importance of β-ketone reduction following *N*-dealkylation. The low formation of M2 suggests that NEP ketone reduction at the β-carbon is not favored, possibly due to a steric effect. This profile was also observed in vitro with RLM incubations. The conjugated metabolites, M7 and M8, were found in greater quantities when compared to M2, M3, and M5, with M8 being slightly more abundant than M7. This may be due to M7 acting as a substrate for the enzymatic machinery, producing M8. This may explain M8’s predominance, despite M7 being an initial product of NEP metabolism. RLM experiments showed that M7 declines progressively as M8 becomes detectable over 1 h of incubation.^14^ Therefore, given our 8 h exposure period, it is plausible that M8 accumulates to higher levels than M7. Phase II metabolites were not observed in water tanks due to an enzymatic hydrolysis step in sample preparation, transforming all conjugated products into their nonconjugated forms for improvement in measurements.

In vitro studies have demonstrated that NEP exhibits substantial neurotoxic effects,^32^ exceeding those demonstrated by similar compounds such as methcathinone, *N*-ethylcathinone, and buphedrone.^33^ Zebrafish exposures to synthetic cathinones also observed stimulant-like effects, locomotors alterations, and withdrawn symptoms, indicating that these substances can cross the blood–brain barrier (BBB) and reach the nervous tissue.^34,35^ Previous studies have also shown that synthetic cathinones, such as methcathinone, can easily cross the BBB.^36,44^ Interestingly, descriptions of neurotoxicity regarding these drugs suggest mechanisms of action that may impair BBB integrity.^32^ *N*-ethyl substituted cathinones, such as NEP, have lower BBB permeability than their *N*-methyl analogs, as previously demonstrated in different animal models. Additionally, the elongation of the alkyl side chain seems to reduce the ability to cross this barrier.^25^ Although these findings indicate that specific NEP structural features negatively affect BBB permeability, they do not exclude its presence in the brain.^25^ Moreover, synthetic cathinones with a propyl side chain have exhibited high potency, suggesting that their toxicity is not fully related to BBB permeability.^37^

Metabolic studies on NEP are scarce in the literature, making this data essential for understanding the drug’s toxicology. To our knowledge, our group was the first to characterize NEP metabolism using microsomal approaches.^14^ While in vitro studies have highlighted unprecedented aspects of NEP metabolism, complementary studies are needed to provide further and comprehensive information. For the first time in the literature, synthetic cathinone’s metabolites were described in brain tissues. The zebrafish blood–brain barrier (BBB) shares structural and functional similarities with the mammalian barrier, including tight junction proteins and efflux transporters, but it has been reported to be comparatively more permeable, particularly at early developmental stages.^38^ Despite this higher permeability in larvae and juveniles, the present ZWT model was conducted in adult zebrafish, in which the BBB is more consolidated and functionally restrictive. This could make difficult the passage of large and polar substances, such as phase II metabolites. Nevertheless, previous studies indicated that several enzymes expressed in the mammalian BBB, such as CYP450 and UGTs, are also present in zebrafish brains. These proteins play a protective role, metabolizing and degrading potentially toxic substances that could damage the brain.^39–41^ Therefore, these data support the hypothesis that the phase I and II metabolites identified in zebrafish brain tissue may originate from local metabolism mediated by enzymes expressed within the CNS.

There were several parallels between in vitro and zebrafish models, including the identified metabolites and their formation ratios. In particular, RLM showed a considerable resemblance to the metabolites found in zebrafish brain, including the predominance of the NEP *N*-dealkylated derivative, the importance of the β-ketone reduction following *N*-dealkylation, and the potential time-dependent formation of M8 from M7. Other studies regarding the metabolism of similar synthetic cathinones have highlighted the importance of reactions such as *N*-dealkylation, β-ketone reduction, aromatic and side chain aliphatic hydroxylations, and glucuronide conjugations.^21,42–45^

The robustness of the MS-based toxicometabolomic analysis was accessed through PCA of QC samples, ensuring the reliability and reproducibility of the data acquisitions. The PCA plot evidenced the good acquisition of the data due to the close distribution of QC samples centered in the plot. However, no clear separation was achieved in the nonsupervised model, with 52.8% of explained variance within two components. This unclear separation can be related to the high chemical complexity present in the samples. The supervised OPLS-DA model demonstrated clear discrimination between NEP-exposed and control groups, supporting the existence of metabolomic alterations associated with NEP neurotoxicity. However, statistical assessment of the model revealed an *R* ^2^ value of 0.69 and a *Q* ^2^ value of 0.30, indicating absence of predictive power. Robust and predictive OPLS-DA models present *Q* ^2^ values above 0.5, with values closer to 1 indicating high robustness.^46^ The relatively low *Q* ^2^ value in our study suggests potential overfitting, likely influenced by the small sample size. Future studies with larger cohorts are warranted to refine the predictive capacity of multivariate models and enhance our understanding of NEP-induced metabolic perturbations.

Despite these statistic limitations, our findings provide novel insights into the neurotoxic effects of NEP, identifying key metabolic pathways potentially involved in its toxicity. A hallmark of NEP neurotoxicity is its action as a monoamine transporter substrate, leading to excessive synaptic accumulation of dopamine and serotonin, excitotoxicity, oxidative stress, and neuroinflammation.^47,48^ In our study, we annotated significantly upregulated metabolites such as cytidine, propionylcarnitine, l-kynurenine, and adenylyl(3′–5′)­cytidine, and downregulated metabolites, such as PI­(19:1­(9Z)/0:0), besides a potentially unknown metabolite.

The upregulation of cytidine and adenylyl(3′–5′)­cytidine suggests an increased demand for nucleotides, potentially reflecting heightened RNA turnover or compensatory mechanisms to counteract oxidative stress-induced damage to nucleic acids.^49^ This nucleoside has been described as a regulator of neuroexcitatory dysfunctions associated with the neuronal–glial glutamate cycle.^50^ Previous study demonstrated that patients with Alzheimer’s Disease (AD), frontotemporal lobe dementia, and Lewy body disease present increased serum levels of cytidine.^51^ Moreover, other inflammatory diseases and cancer have been associated with higher levels of this nucleoside.^52,53^

Simultaneously, the elevated levels of l-kynurenine highlight a shift in tryptophan metabolism toward the kynurenine pathway, a well-established driver of neurotoxicity and neuroinflammation due to the production of excitotoxic and pro-inflammatory metabolites such as quinolinic acid.^54^ Interestingly, pregnant zebrafish exposed to l-kynurenine presented substantial impairments in neurobehavioral development of adult offspring, demonstrating its potential role as a neurotoxic metabolite.^55^ Despite this plausible association, Gulaj et al. demonstrated that AD patients do not exhibit significant increase in l-kynurenine levels when compared to healthy individuals, even though upregulated concentrations of its toxic metabolic, quinolinic acid, are substantially higher.^56^ Nevertheless, further investigation regarding how modulations of l-kynurenine and its metabolites levels can contribute to neurotoxicity remain unclear, since both positive and negative modulations have been associated with neurological conditions.^57,58^

In parallel, the observed increase in propionylcarnitine implicates mitochondrial and energetic dysfunction, as disruptions in fatty acid oxidation and citric acid cycle intermediates are hallmarks of neurological disorders and neurotoxicity.^59^ Impaired mitochondrial function and energetic failure, exacerbated by excessive neurotransmitter accumulation, leads to elevated oxidative stress and intracellular calcium dysregulation, ultimately contributing to neuronal damage and neuroinflammation.^60,61^ An animal model of neurotoxicity, using phenobarbital (PHB) in adult male Sprague–Dawley rats showed a positive correlation between propionylcarnitine concentrations in hippocampus and frontal lobe and the PHB dosage.^59^ Moreover, spatial-lipidomics using matrix-assisted laser desorption/ionization-mass spectrometry revealed upregulated levels of acylcarnitines in an animal model of Parkinson’s disease (PD).^62^

The downregulation of the glycerophosphatidylinositol PI 19:1­(9Z)/0:0 further reinforces this neurotoxic cascade, as these lipids play critical roles in maintaining membrane integrity, intracellular signaling, and synaptic plasticity.^63,64^ The depletion of this class essential phospholipids class may reflect heightened membrane turnover due to oxidative stress-induced lipid peroxidation, further compromising neuronal stability and triggering microglial-mediated neuroinflammation mechanisms.^65,66^ Studies conducted in rats and humans have described imbalances in phosphatidylinositol and lysophosphatidylcholine levels associated with neurological conditions associated with dementia and cognitive impairments, such as global cerebral ischemia and AD.^67,68^ Dyshomeostasis in phosphoinositides have been also associated with other numerous neurodegenerative conditions, such as PD, Huntington’s Disease, and Amyotrophic Lateral Sclerosis.^65^ These effects place a substantial metabolic burden on neurons, as evidenced by the dysregulation of nucleotide, lipid, and amino acid metabolism in our study.

Enrichment analysis revealed that NEP exposure affects critical biochemical networks, including energy metabolism, neurotransmitter biosynthesis, neuroinflammation, nucleotide metabolism, and lipid homeostasis. Interestingly, this study purposes and correlates the administration of a synthetic cathinone and dysfunctions in energetic metabolism. Alterations in leucine, isoleucine, and valine metabolism, as well as disruptions in amino acid metabolism and derivatives, may suggest potential imbalances in neurotransmitter synthesis and function, as well as neuronal energy supply.^69–71^ Dysfunction in pathways associated with energy failure is well-documented in neurodegenerative disorders and drug-induced neurotoxicity, reinforcing the relevance of our findings.^72–76^

Mitochondrial dysfunction, a hallmark of neurotoxicity, can be deflagrated through excessive neurotransmitter accumulation, leading to increased calcium (Ca^2+^) influx, oxidative stress, and mitochondrial permeability transition pore opening.^77^ Notably, alterations in mitochondrial dysfunction were already demonstrated in previous reports on synthetic cathinones toxicity.^77^ Our results corroborate these mechanisms, as alterations in mitochondrial function-related pathways, including citric acid cycle disruption and β-oxidation deficiencies, were among the most significantly affected processes. Furthermore, enrichment analysis focused on enzyme activity alterations, displaying perturbations particularly on mitochondrial complexes and lipid metabolism, highlighting the disruption of neuronal bioenergetics and structural integrity. Previous studies have shown that mephedrone leads to reduced ATP production and increased reactive oxygen species (ROS) generation, further exacerbating neurotoxicity.^78–80^ Similarly, bovine brain microvessel endothelial cells treated with methylenedioxypyrovalerone (MDPV) showed an increase in ROS production, producing a disruption of the endothelial cells of the BBB.^81^

The strong enrichment of pathways related to pyrimidine metabolism underscores the impact of NEP on the zebrafish brain, potentially highlighting impairments of nucleic acid turnover and cellular energy balance. Disruptions in pyrimidine metabolism have been implicated in neurodegenerative conditions, as they can affect DNA and RNA integrity, the biosynthesis of phosphatidylcholine (PC) and phosphatidylethanolamine (PE), impair cellular repair mechanisms, and alter neuronal function.^82,83^ Disorders have been linked to such a condition, cognitive deficits, excitotoxicity, and neuroinflammation being the most reported.^82^ Taken together, these data support the hypothesis that NEP exposure may lead to neuronal damage through similar mechanisms.

Additionally, pathways related to SLC transport disorders, SLC-mediated transmembrane transport, and disorders of transmembrane transporters were significantly enriched, suggesting disruptions in essential membrane transport mechanisms that could contribute to impaired neuronal homeostasis, energetic metabolism, and neurotransmitter dysregulation.^84,85^ Previous studies have demonstrated that synthetic cathinones such as MDPV could diminish the expression of glutamate transporter GLT-1, increasing the concentration of this amino acid in the synapse and promoting excitotoxicity via excessive Ca^2+^ influx.^32^

Likewise, phospholipid dysregulation, particularly related to the glycerophosphatidylinositide PI­(19:1­(9Z)/0:0), is linked to membrane integrity disruption and neuronal dysfunction.^65^ These findings align with studies demonstrating that synthetic cathinones induce oxidative stress, mitochondrial impairment, and neuroinflammatory responses, ultimately compromising neuronal viability.^77^ Neuroinflammation has been widely associated with psychostimulants drug’s neurotoxic events, inducing the release of pro-inflammatory cytokines and activating microglia.^86–89^ Specifically for synthetic cathinones, the evidence neuroinflammation mechanisms associated with neurotoxicity is still scarce, even though repeated administrations of molecules such as mephedrone, methcathinone, and methylone stimulated astrocyte or microglia proliferation in the frontal cortex, striatum, and hippocampus.^89–91^

Elevated levels of l-kynurenine suggest an imbalance in the kynurenine pathway, potentially leading to excitotoxicity and neuroinflammation. Kynurenine pathway dysregulation is known to shift metabolism toward the production of neurotoxic metabolites such as quinolinic acid, which overstimulates *N*-methyl-d-aspartate (NMDA) receptors, leading to neuronal damage.^92^ These mechanisms may be associated with psychiatric diseases such as depression and schizophrenia.^93^ Pathway analysis further revealed metabolic disturbances commonly associated with neurological and inflammatory conditions including schizophrenia, Canavan disease, and sepsis.

Nevertheless, as critically discussed by Lee et al. (2025), pathway-based interpretation of metabolomics data must be approached with caution. Unlike gene expression data, where transcript levels are often coregulated and pathway coherence is higher, metabolite concentrations are influenced by dynamic flux, transport, tissue-specific compartmentalization, and multiple metabolic origins. Furthermore, most pathway enrichment tools were originally developed for transcriptomics and rely on assumptions that are not necessarily valid for metabolites, such as pathway linearity and site-specific activity. Analytical constraints, including incomplete metabolome coverage and challenges in metabolite annotation, further complicate interpretation. Thus, while our pathway analysis provides useful hypotheses, these results should be considered exploratory and require further validation through targeted biochemical assays and integrative multiomics approaches.^94^

Although the experimental design successfully demonstrated the applicability of zebrafish in toxicometabolomics of novel psychoactive substances, we acknowledge that the limited number of biological replicates restricts the extent to which broad biological conclusions can be drawn. Therefore, the findings should be interpreted primarily as a proof of concept, highlighting the potential of zebrafish as a valuable model for investigating the toxicological effects and metabolic fate of emerging drugs. Future studies with larger sample sizes and expanded experimental conditions are warranted to further validate and generalize these observations. In addition to the statistical limitations related to the multivariate analyses, it is important to acknowledge that this study is only based on metabolomic profiling. Although metabolomics provides valuable insights into biochemical alterations, complementary approaches—such as proteomics and transcriptomics—are necessary to build a more comprehensive and integrative understanding of the mechanisms underlying NEP-induced neurotoxicity. Nevertheless, this study constitutes a significant advance in the field, offering valuable evidence of the metabolic disruptions induced by NEP, once it reveals that NEP exposure perturbs essential metabolic pathways linked to neurotoxicity, reinforcing the need for further investigations to elucidate its long-term effects on neuronal function. These results contribute to the growing body of evidence on synthetic cathinone-induced neurotoxicity and highlight the necessity for regulatory policies addressing the risks associated with these emerging psychoactive substances.

## 4 Conclusions

Herein, we provide a comprehensive characterization of NEP metabolism in a zebrafish model, identifying eight metabolites across exposure to water and brain tissue. Our findings highlight *N*-dealkylation and β-ketone reduction as key metabolic pathways, consistent with previous in vitro studies. The detection of metabolites in brain tissue suggests that the zebrafish nervous system may actively participate in NEP metabolism, corroborating existing evidence of CYP450 and UGT enzymatic activity at the BBB. Furthermore, toxicometabolomic analysis revealed significant metabolic alterations induced by NEP exposure including disruptions in neurotransmitter regulation, lipid metabolism, and mitochondrial function. The identification of altered pathways related to oxidative stress, neuroinflammation, and bioenergetic impairment suggested the neurotoxic potential of NEP, paralleling mechanisms commonly associated with synthetic cathinone toxicity. Although the multivariate model exhibited limited predictive power due to sample size constraints, the observed metabolic shifts provide critical insights into NEP-induced neurotoxicity. Collectively, this study advances our understanding of NEP metabolism and its toxicological implications, particularly within the CNS. The metabolic similarities between zebrafish and mammalian models reinforce the translational relevance of our findings. Future studies with larger sample sizes and complementary in vivo models are warranted to further elucidate the mechanistic basis of NEP neurotoxicity and its potential long-term consequences.

## 5 Materials and Methods

### 5.1 Chemical and Reagents

LC–MS grade acetonitrile, methanol, water, methyl *tert*-butyl ether (MTBE), ammonium formate, and sodium tetraborate were purchased from Sigma-Aldrich (St. Louis, MO, USA). Formic acid was purchased from Scharlab (Sentmenat, Barcelona, Spain). Ultrapure water was obtained from a Mili-Q RG system from Millipore (Burlington, MA, USA). *E. coli* K12 β-glucuronidase was purchased from Roche (Vienna, Austria). Gentest 0.5 M phosphate buffer (pH 7.4) was obtained from Corning (Woburn, MA, USA). 3,4-methylenedioxymethamphetamine-d_5_ (MDMA-*d*_5_) and methamphetamine-d_5_ reference materials were acquired from Cerilliant (Round Rock, TX, USA) and were prepared at 100 and 10 μg/mL, respectively, in methanol for use as internal standards (IS). NEP hydrochloride reference material was obtained from Cayman Chemical (Ann Arbor, MI, USA) and was prepared at 1 mg/mL (0.85 mg/mL free base) in methanol. Red sea-salt was purchased from Red Sea Fish (Houston, TX, USA) and it was used for RW preparation, diluting 1.7 g in 1 L of ultrapure water.

### 5.2 Maintenance of Zebrafish and NEP Exposure

Wild-type adult zebrafish were maintained according to standard protocols at a density of two to three animals per liter in 30–50 L tanks filled with nonchlorinated water, chemically and mechanically filtered.^95^ The temperature was set at 26 ± 2 °C, and the established photoperiod cycles consisted of 10 h of dark and 14 h of light. Food was provided twice a day with commercial fish food (Tetramin, Tetra, Blacksburg, VA, USA) and supplemented with artemia once a day. All conducted experiments described in this study were reviewed and approved by the Ethical Committee for Animal Research for the Universidade Estadual de Campinas (6253-1/2023 and 6253-1­(A)/2023).

NEP exposure was adapted from the ZWT protocol, described by De Souza Anselmo et al. (2017).^96^ NEP work solution was prepared in RW at 100 μg/mL. Six months old male zebrafish (*n* = 40) were randomly transferred to 250 mL tanks containing 200 mL of RW at 30 °C, in a density of eight animals per tank. These tanks were divided into three groups: positive control (*n* = 1)–NEP, without animals; negative controls (*n* = 2)–constituted by 16 animals (eight per group) without the drug; and exposure tanks (*n* = 3)–constituted by 24 animals (eight per group) with NEP addition. All tanks containing the studied drug had a final NEP concentration of 0.5 μg/mL. The established concentration was based on a previous ZWT study regarding similar synthetic cathinones.^21^ The exposure was carried out for 8 h, and 5 mL aliquots were collected and immediately stored at −80 °C until sample preparation every hour.

After exposure, the animals were euthanized by hypothermic shock, transferring them into a mixture of ice and water (5:1, *m*/*m*) for 40 min, until opercular movements ceased. The bodies were then decontaminated using ultrapure water and immediately stored at −80 °C until brain extraction. For brain dissections, zebrafish were thawed at 4 °C and placed on a Petri dish. Using appropriate surgical tools and an optical microscope coupled to a computer, the skulls were resected allowing the collection of brain tissues. The brains were weighed (approximately 5 mg each) and pooled, combining 5 brains per pool, resulting in three pooled-samples for negative control and four pooled-samples for exposed zebrafish. The pooled samples were then stored at −80 °C until sample preparation.

### 5.3 Sample Preparation

The protocol for metabolite extraction from exposure water was also adapted from De Souza Anselmo et al. (2017).^96^ Briefly, 1 mL of each 5 mL aliquot was transferred to a 2 mL polypropylene tube, followed by the addition of 20 μL of 600 ng/mL MDMA-*d*_5_ (IS). For a hydrolysis procedure, 1 mL of a solution containing ultrapure water, 0.5 M phosphate buffer (pH 7.4), and β-glucuronidase from *E. coli* K12 (72:25:3, v/v/v) was added to the sample. The mixture was then vortexed and incubated at 60 °C for 1 h. After hydrolysis, 850 μL of sodium tetraborate buffer (pH 9.0) was added to the samples and the analytes were extracted using 2.5 mL of MTBE. The samples were then vortexed and centrifuged at 4000 rpm for 5 min using a Hettich Universal 320 R (Tuttlingen, BW, Germany). The organic phase was evaporated until dryness under a nitrogen flow using TurboVap LV (Biotage, Uppsala, Sweden). Finally, samples were resuspended in 100 μL of water/methanol (95:5, v/v) LC–MS grade and 4 μL were injected into a LC-HRMS system.

For both metabolites extraction and metabolomics evaluation in brain tissues, four 3 mm zirconium beads were added to the pooled samples along with 250 μL of methanol containing methamphetamine-d_5_ (IS) at a final concentration of 50 ng/mL. Blank samples were also prepared in triplicate by adding only the zirconium beads and methanol containing the IS. Samples were homogenized into a BeadBlaster 24 (Benchmark Scientific, Sayreville, NJ, USA) in four cycles of 5 m/s for 20 s each with an interval of 30 s. The homogenates were then transferred to new polypropylene tubes and 5 μL aliquots from each homogenate were pooled together for QC sample preparation. The homogenates, QC, and blank samples were centrifuged at 12,000 rpm for 10 min at 4 °C, and the supernatant was dried under nitrogen flow. Finally, the samples were resuspended in 80 μL of water/acetonitrile (98:2, v/v) LC–MS grade, and 5 μL of each sample was randomly injected into the LC-HRMS system. To equilibrate the chromatographic system, pooled QC samples were initially injected six times as conditioning quality controls. Additionally, to monitor system stability and robustness, the pooled QC were injected throughout the batch every four samples.

### 5.4 LC-HRMS Data Acquisition

To evaluate NEP metabolites in zebrafish exposure water from the ZWT experiment, a Nexera HPLC chromatographic system coupled to an LCMS9030 quadrupole-time-of-flight (QToF) mass spectrometer (Shimadzu, Kyoto, Japan) was employed, with an electrospray ionization (ESI) source operating in positive mode. Chromatographic separation was performed using a Cortecs T3 C18 column (2.1 mm × 150 mm, 2.7 μm) at 40 °C. The mobile phases consisted of 0.1% formic acid in water (MPA) and 0.1% formic acid in methanol (MPB) at a flow rate of 0.3 mL/min. The elution gradient initialized at 20% MPB for 1 min, followed by a linear increase to 95% MPB over 18 min, maintaining this proportion for 3 min, and then returning to the initial condition of 20% MPB for up to 21.1 min; this proportion was maintained for up to 26 min to re-equilibrate the chromatographic system. Interface temperature was set at 400 °C, as well as nebulizing gas, heating gas, and drying gas flow at 2, 10, and 10 L/min, respectively. Desolvation line and heat block temperature were 250 and 500 °C, respectively, while the interface voltage was 3.5 kV. Data were acquired using data-dependent acquisition mode, using top-20 dependent events over the threshold of 1000 counts. For full scan (MS) acquisition, mass ranged from *m*/*z* 60 to 500, and for the triggered dependent events (MS2) from *m*/*z* 60 to 450, using collision energy (CE) in spread mode ranging from 10 to 40 eV.

To identify NEP metabolites in zebrafish brain tissue and perform a toxicometabolomics evaluation of this substance in the CNS, the same chromatographic and mass spectrometry systems were employed. Chromatographic separation was achieved using a Cortecs T3 C18 column (2.1 mm × 150 mm, 2.7 μm) at 40 °C with 0.1% formic acid in water (MPA) and acetonitrile with 0.1% formic acid (MPC) as mobile phases at a flow rate of 0.4 mL/min. Gradient elution started with 2% MPC for 1 min, ramping linearly to 40% MPC for 1 min, then increasing to 90% MPC over 25 min, and finally achieving 100% MPC by 27.5 min. This proportion was maintained for 3.5 min and then returned to the initial condition over 31.01 min, maintained this proportion for up to 35 min for column re-equilibrium. Data acquisition was performed using data-independent acquisition (DIA) combined with one MS event, all ranging from *m*/*z* 60 to 1000 with CE in spread mode ranging from 10 to 40 eV. A total of 28 DIA events with a mass window width of *m*/*z* 33.6 were set, each with an event time of 36 ms, while the MS event had an event time of 100 ms. All mass spectra were acquired in centroid mode.

Additionally, before any data acquisition, the mass spectrometer was calibrated to ensure the resolution and accuracy for mass measurement. Sodium iodide (Na-(NaI)_5_) was employed as a reference standard for calibration, monitoring *m*/*z* 1971.6144, with a maximum mass error of 1 ppm and a minimum resolution of 30,000.

### 5.5 Data Analysis

To describe and characterize NEP metabolites in exposure water and zebrafish brain tissues, all chromatograms and mass spectra acquired were analyzed using Insight Explore software version 1.0.0.0 (Shimadzu, Kyoto, Japan). Identified ions in MS1 mode with *m*/*z* values corresponding to theoretical structures compatible with primary phase I and phase II metabolism reactions were considered and investigated. Furthermore, MS2 mass spectra were used to assess and interpret the exact mass of fragments relative to the parent ion and compare them to the results in the literature. The criteria for metabolite identification were: (I) precursor ions with a mass error less than 5 ppm; and (II) fragment ions with a mass error less than 15 ppm. To further support the identification of NEP metabolites, their fragmentation profiles were also compared to a previous study.^14^ Additionally, only metabolites characterized by a mass spectrum with at least two ions consistent with their fragmentation mechanisms and identification of the precursor exact mass were considered. To evaluate metabolite formation dynamics, linear regressions were performed using GraphPad Prism 9.0 software (GraphPad software, San Diego, California) considering the ratio of the extracted-ion chromatogram (XIC) area using the exact mass of the identified metabolites over the area of the internal standard.

For toxicometabolomics data analysis, the acquired raw data (lcd) were initially converted to.mzML format using MS Convert tool from ProteoWizard software^97^ and then processed using MS-DIAL version 4.9.221218 ^98^ for peak picking, chromatogram deconvolution, alignment, and integration. The parameters for MS-DIAL processing included an MS1 and MS2 tolerance of 0.05 and 0.08 Da, respectively, for data collection; minimum peak height of 5000 amplitude; mass slice width of 0.2 Da for peak detection; sigma window value of 0.5; and MS^2^ abundance cutoff of 20 amplitude for MS2 deconvolution. For identification, accurate mass tolerances for MS1 and MS2 were 0.01 and 0.05 Da, respectively, with an identification score cut off of 70%. ESI^+^-MS/MS database from authentic standards database containing 324,191 records were applied for metabolites identification, considering [M + H]^+^, [M + Na]^+^, [M + NH_4_]^+^, and [M + K]^+^ adducts. For data alignment, the RT and MS1 tolerance were 0.25 min and 0.05 Da, respectively. MS-FINDER version 3.52 was employed to enhance metabolite annotation.^99^ Mass tolerance settings for MS1 and MS2 were defined as 0.05 and 0.08 Da, respectively, with a relative abundance cut off of 20%. The determination of molecular formulas was performed by considering elements such as carbon, hydrogen, oxygen, nitrogen, sulfur, and phosphorus. The following local databases were employed for metabolites annotation: HMDB, Urine, Saliva, Feces, Serum, CSF, SMPDB, LipidMAPS, BMDB, ChEBI, T3DB, and PubChem. The setting “Only use when there is no query in local DBs” for PubChem Online and MINEs (Metabolic In Silico Network Expansions) was selected. Formula candidates matching any local structure databases underwent further investigation. The prioritization process followed ranking formula candidates in descending order of significance and a subsequent evaluation of associated structure candidates based on relevance or confidence level. Additional mass spectrum databases such as Human Metabolome Database (HMDB)^100^ and MassBank^101^ were employed for enhanced metabolite annotation. Finally, metabolites were identified and characterized using a 4-level confidence system for high-resolution mass spectrometry analysis, adhering to the described parameters.^28,29^ Processed data were manually reviewed and curated to eliminate background and blank noises, as well as all NEP-related features (isotopes, adducts, and metabolites), and then exported as a matrix for data filtering. In this step, QC features with a coefficient of variation > 30% were excluded from the subsequent statistical analysis. Missing values initially reported as “0” were converted to “NA”. Only features present in at least 75% of the QCs and 60% of the studied groups were maintained. The data matrix was publicly shared at the MS Interactive Virtual Environment (MassIVE) repository with registry code MSV000098732. The data can be accessed in ftp://massive-ftp.ucsd.edu/v10/MSV000098732/. The processed and filtered data were then uploaded to Metaboanalyst 6.0 ^102^ Data were normalized by the sum of total area, transformed by log_10_, and auto scaled for further statistical analysis. Univariate analyses included *t*-test, considering significant features with *p*-value <0.05, and differentially expressed with a FC ≥ 2.0 and FC ≤ 0.5 considering exposed/nonexposed area ratio. Multivariate analyses were comprised by PCA and OPLS-DA, with features demonstrating Variable Importance on Projection (VIP) score ≥2.0 considered statistically relevant for model discrimination between groups. Furthermore, the area under the curve (AUC) of the receiver operator characteristics (ROC) curve of the selected variables was also employed to evaluate their prediction power.

Metabolic pathway and enrichment analyses were also realized using Metaboanalyst 6.0. Pathway analysis was set for Scatter plot visualization, hypergeometric test enrichment method, topology measurement using relative-betweenness centrality, selecting a pathway library from *D. rerio* based on KEGG pathway database. Enrichment analysis was performed setting pathway (RaMP-DB), disease (blood), and predicted dysfunctional enzymes-based libraries.

## Supplementary Material



## Data Availability

All data presented in this study are available in this article. These data were also part of the master’s thesis of the principal investigator Alexandre Barcia de Godoi (A.B.G).

## Notes

Nonlinear regression curves using NEP over internal standard (IS) area ratio in (A) positive control and (B) exposure tanks over the 8 h incubation period; tandem mass spectra of NEP and its identified phase I (M1-M5) and phase II metabolites (M7 and M8) found in zebrafish brains acquired using LC-HRMS in positive mode; tandem mass spectra of NEP and its metabolites (M1, M3, and M6) found in the exposure water acquired using LC-HRMS in positive mode; NEP and metabolites fragments chemical structures proposals; substituents were included in chemical structures at *N*-terminal amine (R1), benzenic ring (R2), α-aliphatic side chain (R3), β-carbon ethylamine moiety (R4); NA; not applicable, Et: ethyl, OH: hydroxyl group, Ket: ketone moiety; and NEP and its metabolites identified in extracts of zebrafish brain and exposure water after 8 h exposure (Table 1) (PDF)
