# Supplementary material for: Neurotoxicity-Based Toxicometabolomics of N‑Ethyl Pentedrone Using Zebrafish as an In Vivo Model
Source: ACS Omega. 2025 Oct 18;10(42):50626–42. doi: 10.1021/acsomega.5c08710 (PMC12573002; doi:10.1021/acsomega.5c08710)
Supplement: Supplementary file 1 [file ao5c08710_si_001.pdf]

Supporting information for

Neurotoxicity-based toxicometabolomics of *N*-ethyl pentedrone using zebrafish as an *in vivo* model

Alexandre B. Godoi[1,2], Leonardo C. Rodrigues[1,2], Matheus F. Alves[3,4], Viviane C. Fais[2,5], Claudia V. Maurer-Morelli[2,5], Jose L. Costa[1,6*]

1. Campinas Poison Control Center, Universidade Estadual de Campinas (UNICAMP), Campinas, SP, Brazil
2. School of Medical Sciences, Universidade Estadual de Campinas (UNICAMP), Campinas, SP, Brazil
3. Leibniz Institute of Vegetable and Ornamental Crops (IGZ), Theodor-Echtermeyer-Weg 1, 14979, Großbeeren, Germany
4. Institute of Biodiversity, Ecology, and Evolution (IBEE), Friedrich Schiller University Jena, Dornburgerstraße 159, 07743, Jena, Germany
5. Laboratory of Zebrafish, School of Medical Sciences, Universidade Estadual de Campinas (UNICAMP), Campinas, SP, Brazil
6. Faculty of Pharmaceutical Sciences, Universidade Estadual de Campinas (UNICAMP), Campinas, SP, Brazil

*Corresponding author: josejlc@unicamp.br; Tel.: +55-19-3531-7232

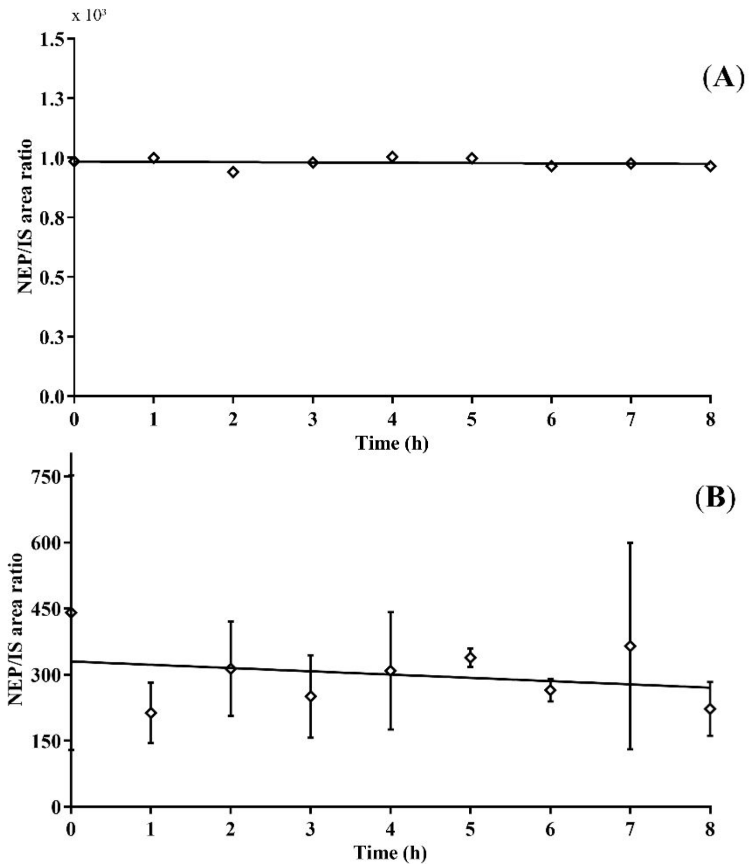

Figure S1. Nonlinear regression curves using *N*-ethyl pentedrone (NEP) over internal standard (IS) area ratio in (**A**) positive control and (**B**) exposure tanks over the 8-hour incubation period.

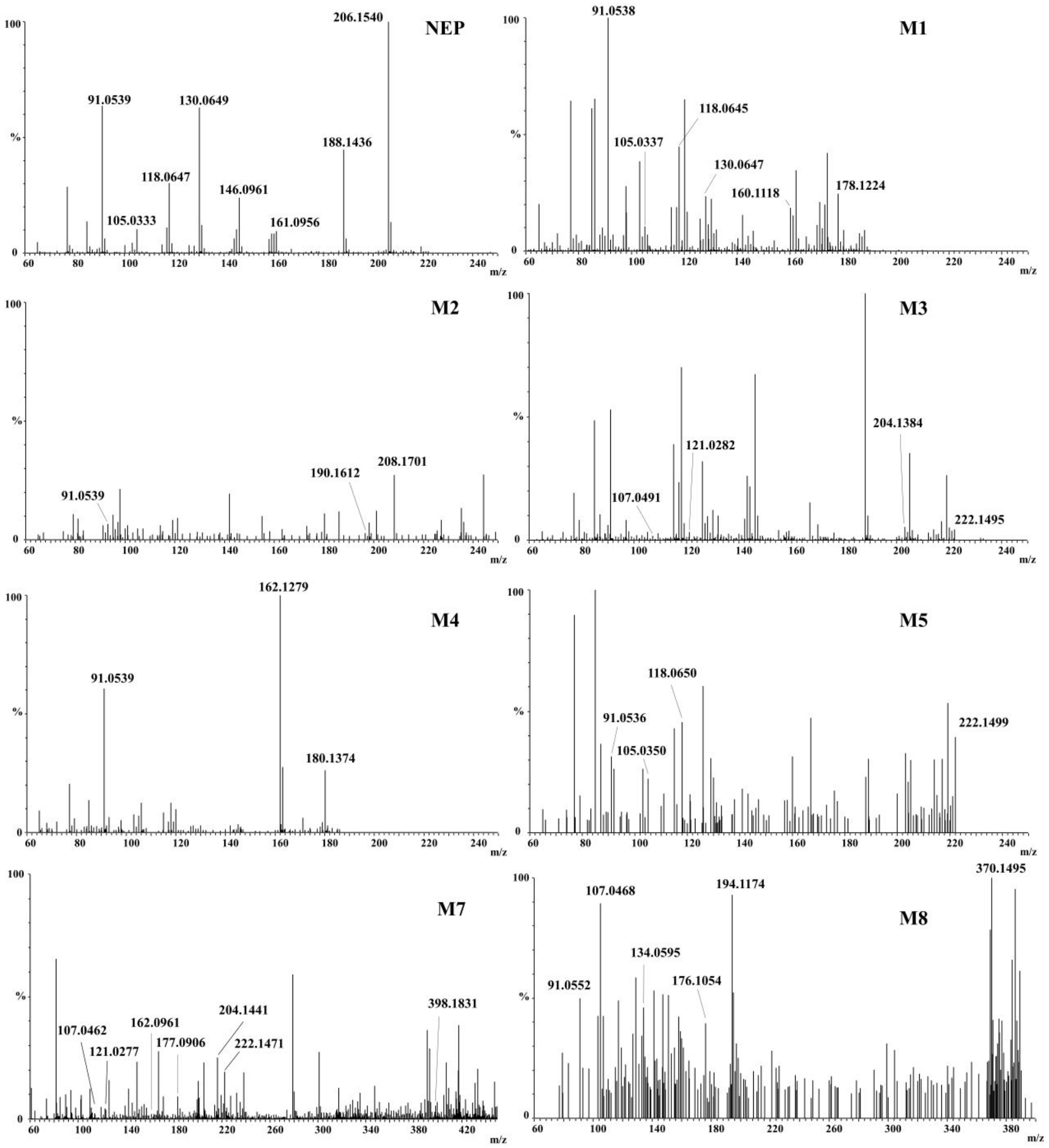

Figure S2. Tandem mass spectra of *N*-ethyl pentedrone (NEP) and its identified phase I (M1-M5) and phase II metabolites (M7 and M8) found in zebrafish brains acquired using LC-HRMS in positive mode.

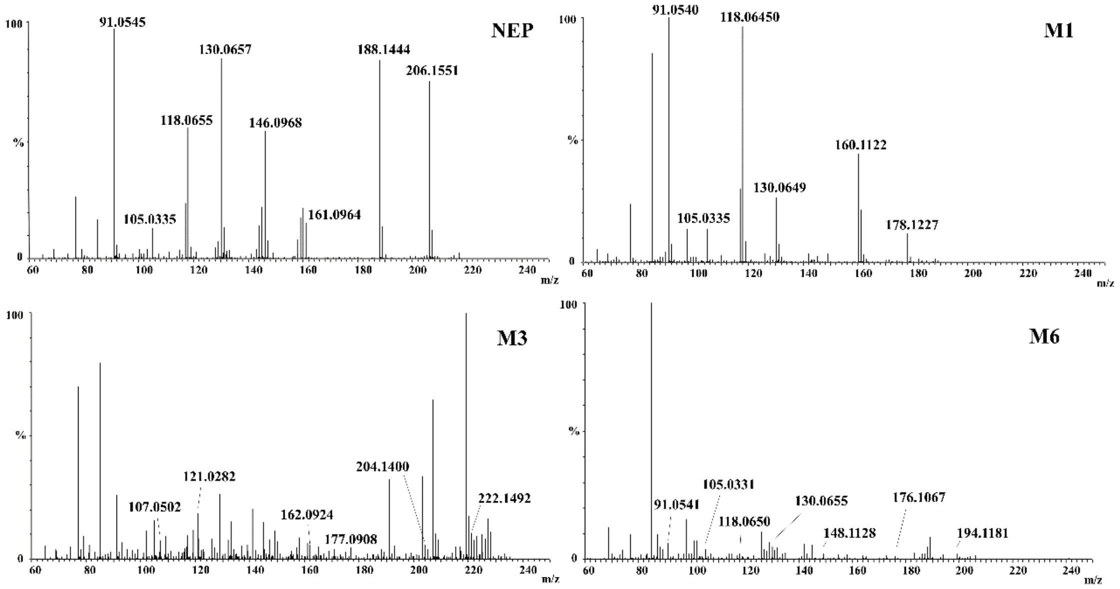

Figure S3. Tandem mass spectra of *N*-ethyl pentedrone (NEP) and its metabolites (M1, M3, and M6) found in the exposure water acquired using LC-HRMS in positive mode.

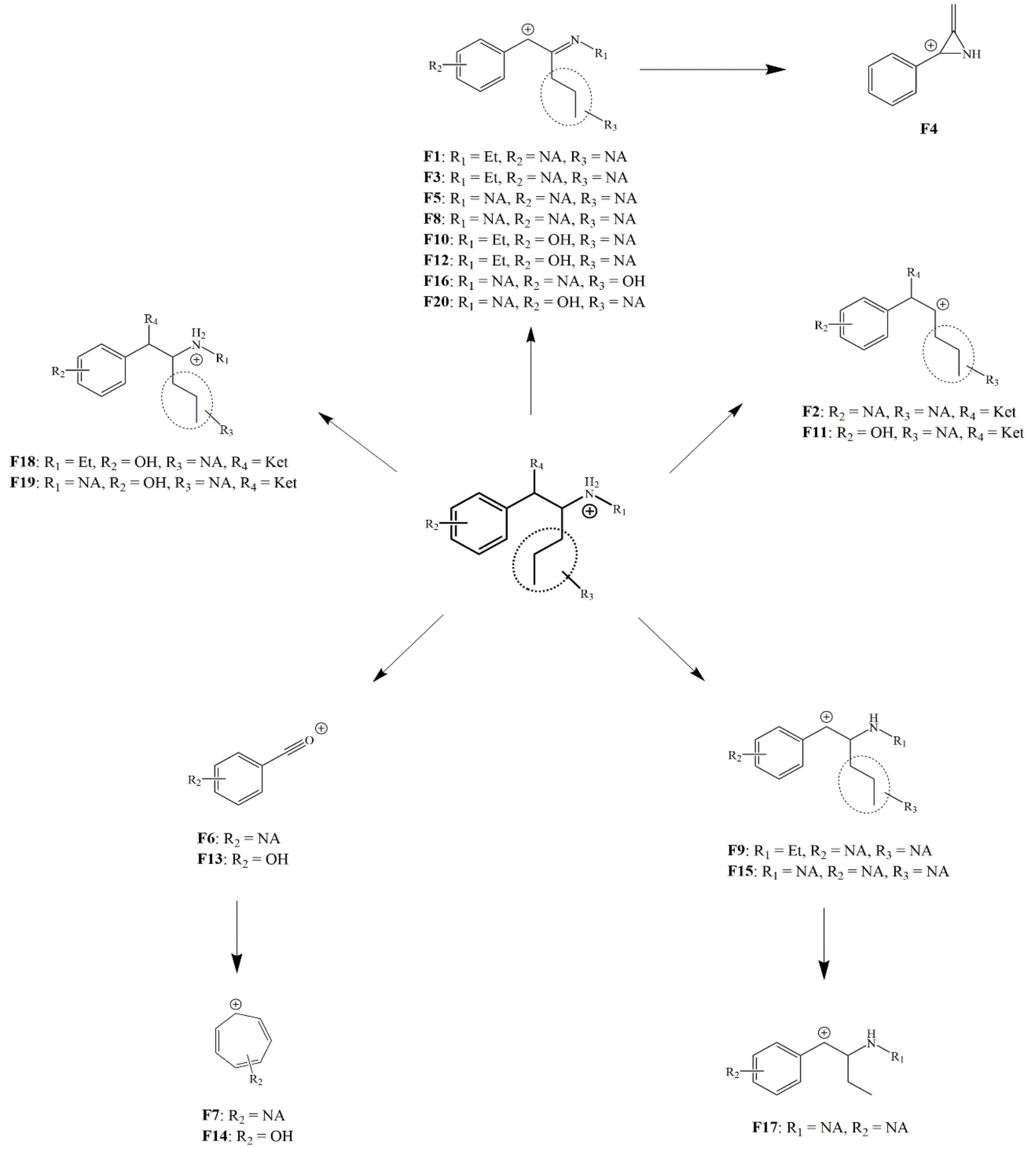

Figure S4. NEP and metabolites fragments chemical structures proposals. Substituents were included in chemical structures at *N*-terminal amine (R_1), benzenic ring (R_2), α-aliphatic side chain (R_3), β-carbon ethylamine moiety (R_4). NA; not applicable, Et: ethyl, OH: hydroxyl group, Ket: ketone moiety.

Table S1. *N*-ethyl pentedrone (NEP) and its metabolites identified in extracts of zebrafish brain and exposure water after 8-hour exposure.

					Zebrafish brain			Exposure water		
**Molecule**	**Metabolism reaction**	**Fragment**	**Molecular formula**	**Theoretical exact mass [M+H]$^+$**	**Measured exact mass [M+H]$^+$**	**Mass error (ppm)**	**Retention time (min)**	**Measured exact mass [M+H]$^+$**	**Mass error (ppm)**	**Retention time (min)**
NEP	-	-	$C_{13}H_{20}NO$	206.1545	206.1540	2.18	3.13	206.1551	-2.96	8.47
		F1	$C_{13}H_{18}N$	188.1439	188.1436	1.93		188.1444	-2.32	
		F2	$C_{11}H_{13}O$	161.0966	161.0956	6.21		161.0964	1.24	
		F3	$C_{10}H_{12}N$	146.0970	146.0961	6.19		146.0968	1.05	
		F4	C_9H_8N	130.0657	130.0649	5.72		130.0657	0.03	
		F5	C_8H_8N	118.0657	118.0647	8.33		118.0655	1.13	
		F6	C_7H_5O	105.0340	105.0333	7.14		105.0335	5.52	
		F7	C_7H_7	91.0548	91.0539	9.39		91.0545	3.35	
M1	*N*-dealkylation	-	$C_{11}H_{16}NO$	178.1232	178.1224	4.60	3.05	178.1227	2.91	8.10
		F8	$C_{11}H_{14}N$	160.1126	160.1118	5.27		160.1122	2.83	
		F4	C_9H_8N	130.0657	130.0647	7.26		130.0649	5.64	
		F5	C_8H_8N	118.0657	118.0645	9.86		118.0650	6.13	
		F6	C_7H_5O	105.0340	105.0337	3.14		105.0335	5.52	
		F7	C_7H_7	91.0548	91.0538	10.27		91.0540	8.73	
M2	β-ketone reduction	-	$C_{13}H_{22}NO$	208.1701	208.1701	0.19	3.85	-	-	-
		F9	$C_{13}H_{20}N$	190.1596	190.1612	-8.39		-	-	
		F7	C_7H_7	91.0548	91.0539	9.72		-	-	
M3	Aromatic hydroxylation	-	$C_{13}H_{20}NO_2$	222.1494	222.1495	-0.48	2.83	222.1492	1.05	7.36
		F10	$C_{13}H_{18}NO$	204.1388	204.1384	2.30		204.1400	-5.44	
		F11	$C_{11}H_{13}$O2	177.0916	-	-		177.0908	4.09	
		F12	$C_{10}H_{12}NO$	162.0919	-	-		162.0924	-3.28	
		F13	$C_7H_5O_2$	121.0290	121.0282	6.24		121.0282	6.32	
		F14	C_7H_7O	107.0497	107.0491	5.70		107.0502	-4.30	
M4	*N*-dealkylation + β-ketone reduction	-	$C_{11}H_{18}NO$	180.1388	180.1374	2.38	3.01	-	-	-
		F15	$C_{11}H_{16}N$	162.1283	162.1279	2.43		-	-	
		F7	C_7H_7	91.0548	91.0539	9.28		-	-	
M5	Aliphatic hydroxylation	-	$C_{13}H_{20}NO_2$	222.1494	222.1499	-2.14	3.00	-	-	-
		F5	C_8H_8N	118.0657	118.0650	5.37		-	-	
		F6	C_7H_5O	105.0340	105.0350	-8.85		-	-	
		F7	C_7H_7	91.0548	91.0536	13.01		-	-	
M6	*N*-dealkylation + Aliphatic hydroxylation	-	$C_{11}H_{16}NO_2$	194.1181	-	-	-	194.1181	0.02	9.70
		F16	$C_{11}H_{14}NO$	176.1075	-	-		176.1067	4.54	
		F17	$C_{10}H_{14}N$	148.1126	-	-		148.1128	-1.13	
		F4	C_9H_8N	130.0657	-	-		130.0655	1.41	
		F5	C_8H_8N	118.0657	-	-		118.0650	6.05	
		F6	C_7H_5O	105.0340	-	-		105.0331	8.76	
		F7	C_7H_7	91.0548	-	-		91.0541	6.97	
M7	Aromatic hydroxylation + *O*-Glucuronidation	-	$C_{19}H_{28}NO_8$	398.1815	398.1831	-4.01	3.61	-	-	-
		F18	$C_{13}H_{20}NO_2$	222.1494	222.1471	10.51		-	-	
		F10	$C_{13}H_{18}NO$	204.1388	204.1441	-25.67		-	-	
		F11	$C_{11}H_{13}O_2$	177.0916	177.0906	5.17		-	-	
		F12	$C_{10}H_{12}NO$	162.0919	162.0961	-25.98		-	-	
		F13	$C_7H_5O_2$	121.0290	121.0277	10.29		-	-	
		F14	C_7H_7O	107.0497	107.0462	32.70				
M8	*N*-dealkylation + Aromatic hydroxylation + *O*-Glucuronidation	-	$C_{17}H_{24}NO_8$	370.1502	370.1495	1.79	3.30	-	-	-
		F19	$C_{11}H_{16}NO_2$	194.1181	194.1174	3.52		-	-	
		F16	$C_{11}H_{14}NO$	176.1075	176.1054	12.37		-	-	
		F20	C_8H_8NO	134.0606	134.0595	8.27		-	-	
		F14	C_7H_7O	107.0497	107.0468	27.18		-	-	
		F7	C_7H_7	91.0548	91.0552	-4.67		-	-	
